# Gastrointestinal Disease in Common Variable Immunodeficiency Disorder (CVID): Histological Patterns, Diagnostic Clues and Pitfalls for the Pathologist and Gastroenterologist

**DOI:** 10.3390/jcm14020497

**Published:** 2025-01-14

**Authors:** Lars Velthof, Jeroen Geldof, Marie Truyens, Jo Van Dorpe, Liesbeth Ferdinande, Ciel De Vriendt, Tessa Kerre, Filomeen Haerynck, Triana Lobatón, Anne Hoorens

**Affiliations:** 1 Department of Pathology, Ghent University Hospital, Ghent University, 9000 Ghent, Belgium; lars.velthof@uzgent.be (L.V.); jo.vandorpe@uzgent.be (J.V.D.); liesbeth.ferdinande@uzgent.be (L.F.); 2 Department of Gastroenterology and Hepatology, Ghent University Hospital, Ghent University, 9000 Ghent, Belgium; jeroen.geldof@uzgent.be (J.G.); marie.truyens@ugent.be (M.T.); triana.lobatonortega@uzgent.be (T.L.); 3 IBD Research Unit, Department of Internal Medicine and Pediatrics, Ghent University Hospital, Ghent University, 9000 Ghent, Belgium; 4 Cancer Research Institute Ghent (CRIG), Ghent University, 9000 Ghent, Belgium; 5 Department of Hematology, Ghent University Hospital, Ghent University, 9000 Ghent, Belgium; ciel.devriendt@uzgent.be (C.D.V.); tessa.kerre@uzgent.be (T.K.); 6 PID Research Laboratory, Department of Pediatric Immunology and Pulmonology, Centre for Primary Immunodeficiency Ghent (CPIG), Jeffrey Modell Diagnosis and Research Centre, Ghent University Hospital, Ghent University, 9000 Ghent, Belgium; filomeen.haerynck@uzgent.be

**Keywords:** histopathology, common variable immunodeficiency disorder (CVID), inflammatory bowel disease (IBD)

## Abstract

**Background/Objectives**: Gastrointestinal diseases are a major cause of morbidity in common variable immunodeficiency disorder (CVID), clinically often mimicking other conditions including celiac disease and inflammatory bowel disease (IBD). Hence, diagnosis of CVID remains challenging. This study aims to raise awareness and highlight histopathological clues for CVID in intestinal biopsies, emphasizing diagnostic pitfalls for the pathologist/gastroenterologist. **Methods**: We reviewed 63 (18 duodenal, 23 ileal, 22 colonic) biopsies and case histories from seven CVID patients, obtained over a 31-year period, with attention to active inflammation, intraepithelial lymphocytes, plasma cells, lymphoid hyperplasia, crypt/villous architecture, subepithelial collagen, apoptosis, granulomas, and infections. Clinical information of 41 pathology requests was reviewed. **Results**: Gastrointestinal symptoms were variable. Histological features included IBD-like (3/7), celiac disease-like (2/7), graft-versus-host disease (GVHD)-like (2/7), lymphocytic sprue/colitis-like (3/7), collagenous colitis-like (2/7), and acute colitis-like (4/7) patterns, often overlapping (2/7) and/or changing over time (3/7). Lymphoid hyperplasia was seen in 3/7 patients; 1/7 had giardiasis; and 5/7 had few plasma cells, usually only in part of the gut (3/5). Clinical information of 12/41 (29%) pathology requests mentioned known/suspected CVID, despite being known in 33/41 (80%). **Conclusions**: Clinical/histological features of CVID in the gut are diverse, often mimicking IBD, microscopic colitis, celiac disease and/or GVHD, hence the importance of adequate clinical information. Some histological features are atypical of these established entities and may indicate CVID, as may overlapping/changing histological patterns and/or few plasma cells in part of the gut. Awareness of the heterogenous clinical presentation and histopathological indicators of CVID may improve diagnosis.

## 1. Introduction

Common variable immunodeficiency disorder (CVID) is a primary immunodeficiency (PID) characterized by low levels of serum immunoglobulins and an impaired antibody response [1,2]. The disease was first described by Janeway et al. in 1953 [3]. It is the second most common PID following selective immunoglobulin (Ig) A deficiency and is the most common symptomatic PID with a prevalence ranging from 2 to 4 in 100.000 [4,5,6,7,8]. It usually presents between the second and fourth decade, although diagnosis of the disease is often delayed by 6 to 8 years, potentially resulting in life-threatening complications such as infections or malignancies [6,8,9,10]. The mortality rate of CVID ranges between 23% and 27%, with a median life expectancy of 54 years [11,12].

The diagnosis of CVID is confirmed by both laboratory investigations showing a significant reduction in serum levels of at least two immunoglobulins (IgG and IgA and/or IgM), at least two standard deviations (SDs) below the age-specific mean values, and an impaired or absent specific antibody production in response to protein or carbohydrate vaccines, or a recent infection, in the absence of secondary causes of hypogammaglobulinemia [2,9,13]. The hypogammaglobulinemia in CVID results from the failure of B-cells to differentiate into plasma cells. However, the exact pathogenesis of CVID is poorly understood and other mechanisms such as T-cell dysregulation and defective cytokine production may also play a role [6,8,14,15,16].

CVID patients mostly present with chronic or recurrent sinopulmonary infections such as pneumonia, bronchitis and/or sinusitis, which are often one of the first signs of the disease. However, numerous other clinical manifestations have been reported, including malignancies, lymphoproliferative disorders, autoimmunity and/or gastrointestinal (GI) disease, the latter of which may be the initial presentation [7,8,10,17,18].

GI complications are the second most prominent cause of morbidity in patients with CVID, after respiratory complications [4]. The incidence of GI manifestations in CVID ranges from 20 to 60%, with diarrhea being the most frequently reported GI symptom [1,4,10,18]. GI manifestations in CVID patients can be due to infections or noninfectious causes [4]. The clinical presentation may closely resemble inflammatory bowel disease (IBD), both Crohn’s disease (CD) and ulcerative colitis (UC), and other GI conditions such as celiac disease, Whipple’s disease, lymphocytic colitis, collagenous colitis, and/or graft-versus-host disease (GVHD) [1,7,8,12,19,20,21,22,23,24,25,26]. In 6% to 10% of patients, an IBD-like presentation is seen [4,8,18], mostly after the diagnosis of CVID [26], and up to 35% of CVID patients with GI manifestations are initially diagnosed with IBD [8,12].

Treatment for CVID involves intravenous IgG (IVIg) or subcutaneous Ig (SCIg) administrations, which reduce the risk of infections but only have a limited effect on other complications such as noninfectious enterocolitis, in part because the IgG in these preparations cannot reach the lumen of the bowel [1,4,15,26,27,28,29].

This study aims to contribute to higher awareness of CVID and highlight histopathological clues for CVID in intestinal biopsies, while emphasizing diagnostic pitfalls for both the pathologist and gastroenterologist. Higher awareness of the heterogenous clinical presentation and better recognition of histopathological indicators of CVID may contribute to earlier diagnosis and treatment, thereby avoiding potentially life-threatening complications.

## 2. Materials and Methods

CVID patients who were identified as having GI manifestations (either by the clinician or through medical records/archived pathology records) and had GI biopsies taken at our institution (Ghent University Hospital, Ghent, Belgium) were eligible for inclusion in this study. Diagnosis of CVID was based on reduced (>2 SD below age-specific mean) serum IgG, IgA and/or IgM levels and impaired or absent specific antibody responses (or impaired B-cell maturation) in the absence of secondary causes of hypogammaglobulinemia [2,9,13].

After approval (14 July 2023) by our institutional ethics committee (reference number CR-2023-0001), we identified seven eligible patients by contacting clinicians and searching archived pathology records. All patients provided written informed consent. Initially, eight patients were identified, but one patient, initially thought to have CVID and later diagnosed with combined immunodeficiency disorder (CID), was excluded from this study.

Medical records including case histories, clinical records, endoscopy reports and pathology records were reviewed for information on clinical presentation, (differential) diagnosis and any confirmed infections. Available biopsy samples were retrieved from the pathology archives of our institution. We also reviewed clinical information of 41 accompanying pathology requests for mention of known/suspected CVID.

Biopsies were obtained between 1 January 1993, and 30 November 2024, and were of esophageal, gastric, duodenal, ileal or colonic origin. In this study, only biopsies from the bowel (duodenum, ileum and/or colon) were included. Because of similar appearance from site to site within one organ, each set of biopsies from the same organ upon the same occasion was considered as a single sample (e.g., a set of biopsies taken at different sites within the colon was regarded as a single colonic biopsy because of similar histological appearance).

All routine hematoxylin and eosin (HE)-stained slides and immunohistochemical stainings, available at our institution, were reassessed with particular attention to features indicative of CVID. Based on an extensive literature review, these features included distorted crypt and/or villous architecture, active inflammation, lymphoid hyperplasia (LH), increased apoptosis, intraepithelial lymphocytosis, subepithelial collagen deposition, granulomas, a paucity of plasma cells and presence of any infectious causes.

Shortened crypts, destroyed crypts and irregularly shaped or branching crypts were considered features of crypt distortion. In the duodenum, villous atrophy was assessed. Neutrophilic inflammation in the mucosa (cryptitis and/or crypt abscesses) was considered active inflammation. Presence of multiple reactive lymphoid follicles with germinal centers was considered LH. Apoptosis was graded on routine HE slides and ≥2 crypt apoptotic bodies per 10 to 15 crypts was considered abnormal [12]. To assess the number of intraepithelial lymphocytes (IELs), CD3 [2GV6 clone, Ventana (Roche, Basel, Switzerland)] immunohistochemistry (IHC) was performed and >5 IELs per 100 surface epithelial cells was considered abnormal (mild/moderate intraepithelial lymphocytosis) [30], whereas >20 IELs per 100 surface epithelial cells was considered a severe increase. Tenascin-C [T2H5 clone, OriGene (Rockville, MD, USA)] IHC had been performed to evaluate subepithelial collagen deposits. A subepithelial collagen layer of ≥3 µm was considered abnormal [30], and > 10 µm was considered substantial thickening. Plasma cells were assessed using multiple myeloma oncogene-1 (MUM1) [MUM1p clone, Dako, Agilent (Santa Clara, CA, USA)] and CD138 [MI15 clone, Dako, Agilent (Santa Clara, CA, USA)] IHC. Any noticeable decrease (even if only focal) in the number of lamina propria (LP) plasma cells compared to normal GI biopsies was considered abnormal. In cases with active inflammation, cytomegalovirus (CMV) [CCH2 and DDG9 clones, Dako, Agilent (Santa Clara, CA, USA)] IHC was performed to rule out CMV enterocolitis.

All immunohistochemical stains were performed in the laboratory of our institution with a Roche (Basel, Switzerland) Ventana immunostainer using standard immunohistochemical methods with the inclusion of appropriate positive and negative tissue controls. All slides were evaluated using an Olympus BX41 microscope (Tokyo, Japan).

Histological findings were classified into the following patterns: an IBD-like pattern, a celiac disease-like pattern, a GVHD-like pattern, a lymphocytic sprue/lymphocytic colitis-like pattern, a collagenous colitis-like pattern, and an acute colitis-like pattern. In case multiple patterns were recognized in a biopsy, each of these was listed. Biopsies with histological alterations not fitting within any of the preceding patterns were considered to show no specific pattern. Biopsies not showing any significant histological alterations were classified as normal.

## 3. Results

The medical records and clinical characteristics of seven CVID patients (three males, four females) aged 20 to 62 years (median 49 years) were reviewed. The age at diagnosis of CVID ranged from 11 to 53 years (median 44, IQR 19; mean 36.9). Except for one patient who was diagnosed at age 11, all other patients were diagnosed well beyond adolescence. Clinical data and medical records were available for all patients. All seven patients had a history of recurrent respiratory infections and GI problems. Two patients also had other recurrent infections including recurrent mastitis or skin infections. All patients had been treated with IVIg or SCIg since diagnosis of CVID.

The most common GI manifestation was diarrhea, which was reported in all seven cases. Abdominal discomfort was reported in six cases. Three patients had blood in their stool. Two patients had a history of obstruction and/or constipation. One patient had signs of malabsorption. Upon endoscopy, nodular lymphoid hyperplasia (NLH) was seen in the terminal ileum of two patients. One patient was diagnosed with irritable bowel syndrome (IBS). Two patients were found to have lactose intolerance.

Two patients were first diagnosed with CD (based primarily on IBD-like endoscopic features), before they were eventually found to have CVID. One of these patients was later, after diagnosis of CVID, also thought to have celiac disease (based on endoscopic and histological evidence of villous blunting). However, there was neither histological nor clinical response to gluten withdrawal. One other patient also had a celiac disease-like clinical presentation. However, for this patient, there were no recorded data on the response to gluten withdrawal. Neither patient with a celiac disease-like presentation had serological testing nor human leukocyte antigen (HLA) typing at the time of inclusion in this study.

In two patients, CVID was associated with autoimmune diseases. One patient had spondyloarthropathy and one patient had both autoimmune hemolytic anemia (AIHA) and idiopathic thrombocytopenic purpura (ITP). Two patients presented with neoplasia: one patient was diagnosed with a chondrosarcoma before diagnosis of CVID and one patient had developed Burkitt lymphoma after diagnosis of CVID. One patient presented with benign lymphadenopathies. One patient had a genetic disorder; this patient had Ehlers–Danlos syndrome.

Out of the 41 pathology requests accompanying reviewed biopsies, only 12 (29%) mentioned suspected or known CVID in the clinical information; despite this, in 33 (80%) of these pathology requests, this information was on file in medical records at that time.

For all patients, biopsy samples were available and a total of 63 biopsies were reviewed. These included 18 duodenal biopsies, 23 ileal biopsies, and 22 colonic biopsies. Metachronous sets of biopsies from the same site were available since all patients underwent multiple endoscopies. The clinical characteristics of patients, endoscopy findings and histopathological findings in the duodenum, ileum and colon are shown in detail in Table A1 (Appendix A). Histopathological findings in patients are summarized in Table 1.

**Figure 1:**
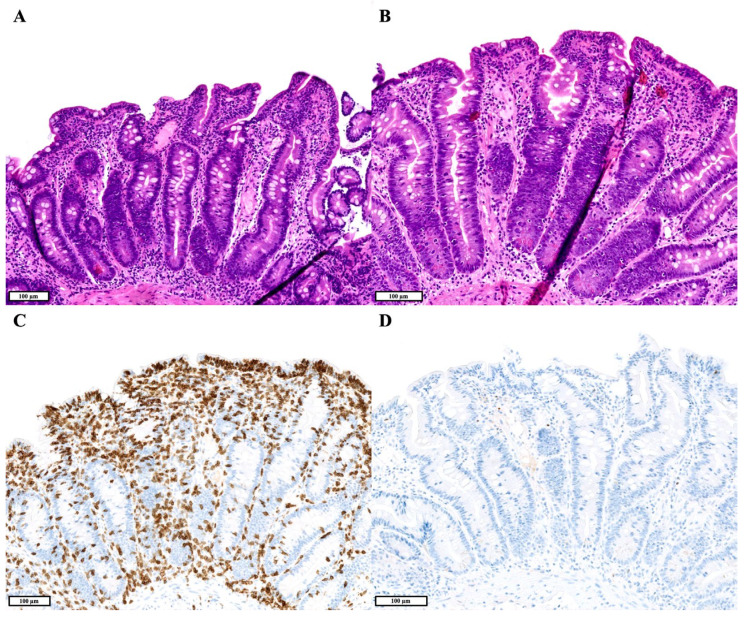
(**A**,**B**). Hematoxylin and eosin staining showing duodenal mucosa with a celiac disease-like pattern in a patient with common variable immunodeficiency disorder (CVID). There is subtotal/total villous atrophy and marked intraepithelial lymphocytosis, as well as crypt hyperplasia. (**C**). CD3 immunohistochemistry (IHC) shows an increased number of intraepithelial lymphocytes. (**D**). Multiple myeloma oncogene-1 (MUM1) IHC shows a lack of plasma cells in the lamina propria.

For all patients, duodenal samples were available, and a total of 18 duodenal biopsies were reviewed. In two patients, none of the duodenal samples showed any significant histological alterations. Four patients had a reduced number of plasma cells in their duodenal biopsies, of whom three patients at some point also had an increased number of IELs. Two of these three patients even showed a celiac disease-like pattern with a severely increased number of IELs and villous atrophy, with one of both also showing crypt hyperplasia (Figure 1). The fourth patient had a reduced number of plasma cells with focal foveolar gastric metaplasia and a normal number of IELs. One patient’s biopsies showed a mild/moderate increase in the number of IELs and presence of *Giardia lamblia*, with a normal number of plasma cells. None of the duodenal biopsies showed lymphoid hyperplasia nor active inflammation.

**Figure 2:**
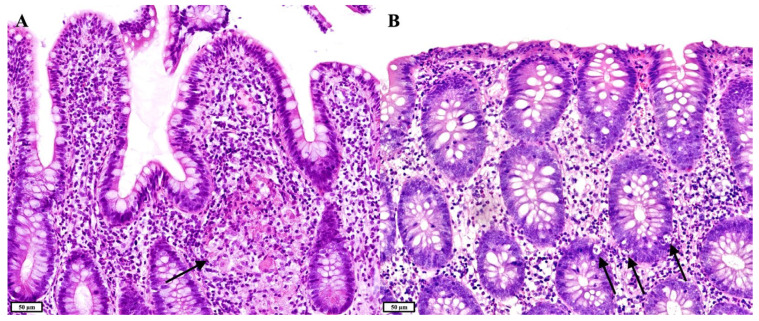
(**A**). Hematoxylin and eosin (HE) staining showing ileal mucosa with active inflammation, a preserved crypt and villous architecture and a granuloma (black arrow) (IBD-like pattern). (**B**). HE staining showing colonic mucosa with active inflammation, a preserved crypt architecture and an increased number of crypt apoptotic bodies (black arrows), mimicking graft-versus-host disease (GVHD) (GVHD-like pattern).

All patients had ileal biopsies taken, and 23 ileal biopsies were reviewed. Two patients at some point had a reduced number of plasma cells in their biopsies. In one of these patients, there was a lymphocytic sprue-like pattern with a severely increased number of IELs, while in the other patient there was an IBD-like (i.e., CD-like) pattern with active inflammation and the presence of a granuloma (Figure 2A). The latter patient had LH and mild/moderate intraepithelial lymphocytosis, respectively, in subsequent ileal biopsies. All other patients had a normal number of plasma cells in their ileal biopsies, with four of these patients at some point having a mild/moderate increase in the number of IELs. Among these four patients, one patient also had LH, and one patient had an infection with Giardia lamblia. Another of these four patients later had an IBD-like (i.e., CD-like) pattern with active inflammation and (pseudo)pyloric gland metaplasia, indicating chronic ileitis. This pattern was also seen in the last patient’s ileal biopsies, where it additionally featured architectural changes (crypt distortion), and occasionally LH was observed.

**Figure 3:**
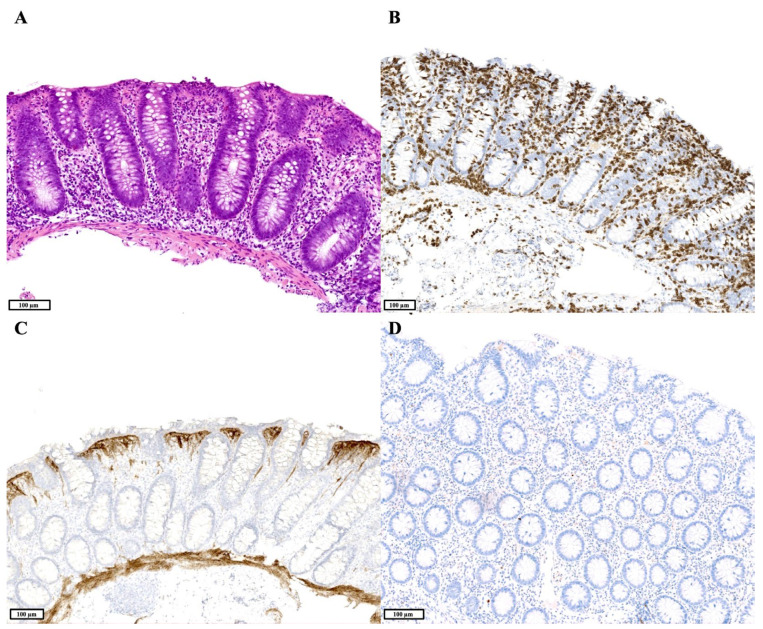
(**A**). Hematoxylin and eosin staining showing colonic mucosa with a preserved crypt architecture, active inflammation and an increased number of intraepithelial lymphocytes. (**B**). Intraepithelial lymphocytosis is confirmed by CD3 immunohistochemistry (IHC) (lymphocytic colitis-like pattern). (**C**). Tenascin-C IHC shows a substantially thickened subepithelial collagen layer (>10 µm) in the colon, mimicking collagenous colitis (collagenous colitis-like pattern). (**D**). Multiple myeloma oncogene-1 (MUM1) IHC shows a lack of plasma cells in the colon.

Colonic biopsies were available for all patients, and a total of 22 sets were reviewed. Six patients’ biopsies at some point showed active inflammation. Four of these patients at some point had an acute colitis-like pattern without architectural changes, while the fifth patient’s colonic biopsies showed an IBD-like pattern with active inflammation and crypt distortion. The sixth patient’s colonic biopsies first showed active inflammation, contributing to the IBD-like (i.e., CD-like) pattern, shaped in part by the concurrent granuloma and active inflammation in the ileum of this patient. Subsequent colonic biopsies of this patient showed an overlapping GVHD-like and lymphocytic colitis-like pattern, with both an increased number of crypt apoptotic bodies (Figure 2B) and a severely increased number of IELs (Figure 3A,B), while later sets of biopsies showed a collagenous colitis-like pattern with only minor active inflammation, a severely increased number of IELs and substantial thickening of the subepithelial collagen layer (Figure 3C). This patient also had a reduced number of plasma cells in multiple colonic biopsies (Figure 3D), which was also seen in one of the four patients with an acute colitis-like pattern. This patient concurrently had a celiac disease-like pattern in the duodenum, a lymphocytic sprue-like pattern in the ileum and an acute colitis-like pattern in the colon. Among the four patients with an acute colitis-like pattern, another patient had previous biopsies showing a GVHD-like pattern with active inflammation, increased apoptosis and mild/moderate intraepithelial lymphocytosis, followed by a collagenous colitis-like pattern with only substantial thickening of the subepithelial collagen layer. The seventh patient’s colonic biopsies showed a lymphocytic colitis-like pattern with severe intraepithelial lymphocytosis and a reduced number of plasma cells, in the absence of active inflammation.

**Figure 4:**
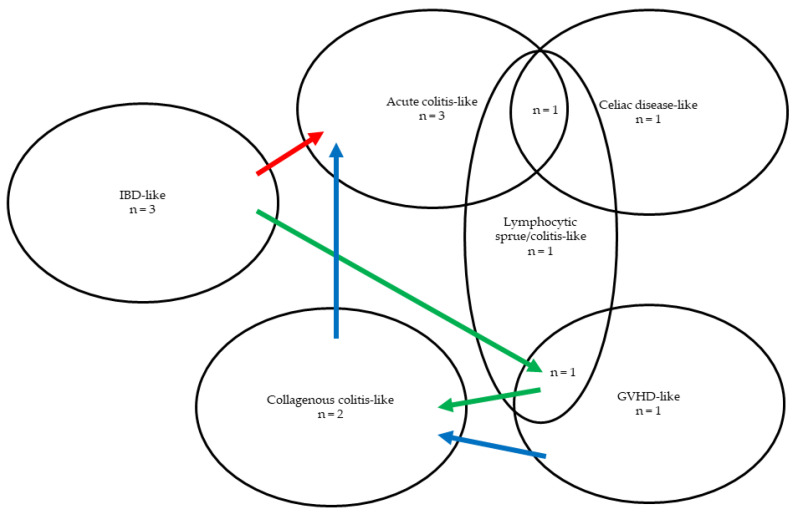
Venn diagram showing overlapping histological patterns (n = the number of patients with a certain pattern). The arrows show evolution of patterns over time, each color of arrow representing a different patient.

An acute colitis-like pattern was seen in four patients. Three patients had an IBD-like (i.e., CD-like) pattern. A lymphocytic sprue-like (1/7 patients) or lymphocytic colitis-like (2/7 patients) pattern was seen in three patients. Two patients had a celiac disease-like pattern. A collagenous colitis-like pattern was seen in two patients, and two patients had a GVHD-like pattern. In two patients, concurrent/overlapping histological patterns were observed: one patient had an overlapping GVHD-like and lymphocytic colitis-like histological pattern in the colon, while another patient had concurrent celiac disease-like, lymphocytic sprue-like and acute colitis-like patterns. In three patients, histological patterns changed over time in consecutive sets of biopsies: the first patient initially had an IBD-like pattern followed by an overlapping GVHD-like/lymphocytic colitis-like pattern and subsequently a collagenous colitis like pattern (Figure 4, green arrows), the second patient had an IBD-like pattern later evolving into an acute colitis-like pattern (Figure 4, red arrow), and the third patient first had a GVHD-like pattern followed by a collagenous colitis-like pattern and an acute colitis-like pattern (Figure 4, blue arrows). LH was seen in three patients and one patient had a Giardia lamblia infection. Overlapping/changing patterns are shown in Figure 4.

Six patients had active inflammation in at least one part of the gut, while five patients had a reduced number of plasma cells in at least one sampled part of the bowel. Of these five patients, only one concurrently had a paucity of plasma cells in both the duodenum and the ileum, as well as in the colon, while in three patients, the paucity of plasma cells was limited to only one part of the gut. One patient had normal plasma cell counts throughout the entire bowel in all biopsies. Six patients had an increased number of IELs in at least one sampled part of the bowel, of whom one patient concurrently had an increased number of IELs in all sampled parts of the bowel. Five patients had a concurrent paucity of plasma cells and intraepithelial lymphocytosis, each in at least one part of the bowel. One patient had neither a paucity of plasma cells nor an increased number of IELs in any of his bowel biopsies.

Except for one patient who had an infection with Giardia lamblia, for the remaining patients, no other infectious agents were identified in any of the biopsies, nor were found by conventional microbiology. In this single patient with diarrhea and abdominal discomfort, Giardia lamblia was identified in both the duodenum and ileum on routine HE-stained slides. CMV IHC was negative in all patients’ biopsies.

## 4. Discussion

CVID usually presents between the second and fourth decade of life [6,8,9,10], as was reflected in this study. It is reported to be associated with lactose intolerance [31], which was the case for two study patients. One of our patients had Ehlers–Danlos syndrome, a condition possibly linked to hypogammaglobulinemia [32]. Additionally, CVID is often associated with autoimmune diseases [1,12,17,22], most commonly ITP and AIHA [26,33]. In this study, one patient had AIHA and ITP, and one patient had spondyloarthropathy. Clinical manifestations of CVID are heterogenous and mainly include recurrent infections, autoimmunity, lymphoproliferation, GI disease and malignancies [17]. In this cohort, all patients presented with recurrent respiratory infections, which are often one of the first signs of the disease and are the most important cause of morbidity [4]. However, GI manifestations are also a major cause of morbidity, with clinical and endoscopic features often mimicking other GI conditions including celiac disease, IBD and microscopic colitis [1,7,8,12,19,20,21,22,23,24,25,26]. In this study, diarrhea was the most common GI manifestation, followed by abdominal discomfort, which is in line with other studies [10]. However, none of the patients in this study had protein-losing enteropathy, a condition that on the one hand can mimic CVID because of serum loss into the bowel lumen and on the other hand can occur in association with CVID [34]. Some patients with CVID may have GI manifestations as their initial presentation [7,18,22]. As such, the gastroenterologist should always consider immunodeficiencies in the differential diagnosis of patients with chronic GI problems, even in the absence of recurrent infections [1], and multidisciplinary immunological work-up (including immunoglobulins and subclass dosing, as well as evaluation of B- and T-cell maturation) should be considered in such patients before starting immunosuppressive therapy. Moreover, genetic screening for PID can be of use in these patients.

There is an increased incidence of IBD in patients with CVID [7], and there is an increased incidence of PID (including CVID) in patients with early-onset IBD [29,35]. However, IBD can be mimicked by a CVID-associated enterocolitis, with distinct histopathological features atypical of IBD [1,7]. Endoscopic features may be indistinguishable from those of IBD and include (longitudinal) ulcers and cobblestone appearance [26]. In the literature, an IBD-like presentation is reported in 6 to 10% of CVID patients [4,8,18], and up to 35% of CVID patients with GI manifestations are initially diagnosed with IBD [8,12], although Albshesh et al. report that the diagnosis of hypogammaglobulinemia more often precedes the diagnosis of IBD-like features [26]. However, this was not observed in our study, where two patients had a CD-like clinical presentation with IBD-like endoscopic features, before eventually being diagnosed with CVID. While one patients’ biopsies showed a true IBD-like pattern, review of the other patients’ biopsies revealed histopathological features that are atypical of IBD and may indicate CVID, including severely increased IELs (lymphocytic colitis-like pattern), increased apoptosis (GVHD-like pattern) and a relative paucity of plasma cells. In typical CD or UC, there is no significant increase in the number IELs, although rarely focal collagenous colitis or lymphocytic colitis patterns have been described in CD [12,36,37]. However, since these are mostly older papers from before 2009, and more recent evidence on this topic is lacking, the question may arise if these are in fact true cases of CD or perhaps unrecognized cases of CVID. Reportedly, some cases of GI inflammation in CVID patients can be diagnosed as true IBD, while in others it is more accurately referred to as IBD-like [8]. However, this topic remains controversial and there is no consensus [8]. Although dysfunctional B-cell differentiation and activation of tumor necrosis factor-alpha (TNFα) play a role in the onset of IBD-like symptoms in CVID patients [26,29,38], the exact pathogenesis remains poorly understood [18,28,29]. CVID-associated enterocolitis can be treated with medication used to manage IBD in immunocompetent patients [8,12,26,39], including biologics [40]. However, although controlling inflammation is crucial, immunosuppressive medications to treat IBD-like symptoms in patients with CVID should be administered carefully [8,12,26,39], preferably after multidisciplinary consultation.

Both the pathologist and the gastroenterologist should be aware that CVID-associated enterocolitis can mimic IBD (both CD and UC), with clinical, endoscopic and histopathological features that closely resemble those of IBD. Hence, care must be taken when a diagnosis of IBD is considered in the context of immunodeficiency and careful attention should be paid to histopathological features atypical of classical IBD (e.g., increased IELs and/or paucity of plasma cells). Moreover, it is of utmost importance that adequate clinical information be provided to the pathologist (i.e., mention of a known/suspected diagnosis of a PID or recurrent infections) to avoid misdiagnosis. In this study, clinical information mentioned suspected or known CVID in only 29% of pathology requests (despite this information being on file in medical records in 80%), complicating the pathologist’s diagnosis.

CVID-associated enterocolitis is not only reported to share histopathological features with IBD but also shares features with lymphocytic colitis, collagenous colitis and colitis associated with GVHD [1,7,12,22]. Byrne et al. first described an association between CVID and collagenous colitis [25], which was later also detailed by other authors [24,38,41]. The intraepithelial lymphocytosis mimicking lymphocytic colitis in CVID is thought to originate from epithelial damage, whereas longer standing disease is thought to cause accumulation of subepithelial collagen, mimicking collagenous colitis [12]. In this study, a collagenous colitis-like pattern was seen in two patients, while a lymphocytic sprue-like/lymphocytic colitis-like pattern was observed in three patients. According to Khodadad et al., CVID-associated colitis can be divided into three distinct patterns, based on histopathological features: (1) a crypt destructive pattern, (2) a non-crypt destructive pattern, and (3) a GVHD-like pattern [1]. The crypt destructive pattern comprises active inflammation with crypt distortion (IBD-like pattern) and the non-crypt destructive pattern encompasses intraepithelial lymphocytosis (lymphocytic sprue-like/lymphocytic colitis-like pattern) and potential thickening of the subepithelial collagen layer (collagenous colitis-like pattern), while the GVHD-like pattern covers an increase in crypt apoptotic bodies (due to viral infections causing an increase in epithelial apoptosis) with eventual loss of crypts [1,12]. The latter can also be seen in the context of an autoimmune colitis when there is no history of an allogeneic transplant; hence, it could also be considered an autoimmune colitis-like pattern. However, to comply with the terminology used in the existing research, we chose to refer to this pattern as a GVHD-like pattern. It has to be noted that Khodadad et al. do not describe an acute colitis-like pattern, as we do. However, since this pattern encompasses cryptitis and/or crypt abscesses (in the absence of signs of chronic inflammation), it can be considered a crypt destructive pattern. Although some authors have described patterns in CVID-associated enterocolitis similar to those described by Khodadad et al. [1,22], van Schewick et al. suggest that bowel histology in CVID patients falls into three different major histological patterns: (1) a celiac disease-like pattern, (2) an IBD-like pattern and (3) NLH [20]. It should be noted that the classification of Khodadad et al. covers only the large intestine [1], while the classification of van Schewick et al. covers both the large and small intestine, as well as the duodenum [20]. However, van Schewick et al. did not consider other patterns such as the GVHD-like pattern [20].

According to our findings it seems that overlap between different histological patterns is possible, and patterns can change over time (e.g., one patient initially had an IBD-like pattern followed by an overlapping GVHD-like/lymphocytic colitis-like pattern and subsequently a collagenous colitis like pattern), which, to the best of our knowledge, has not yet been described in the literature. Evolution in patterns was also reflected in the clinical presentation as most patients presented with different GI symptoms over time (e.g., this patient initially had a CD-like clinical presentation and later presented with chronic watery diarrhea). These overlapping and/or evolving patterns are quite remarkable as they are not to be expected in immunocompetent patients. Hence, this could be another diagnostic clue for the pathologist and gastroenterologist. Since we report many different histological patterns that can overlap and/or change over time, and since neither of the proposed classifications cover the entire spectrum of histological patterns that CVID can mimic, we believe that CVID-associated enterocolitis should not necessarily be divided into the limited pattern groups proposed by van Schewick et al. or Khodadad et al. [1,20]. Instead, we think CVID-associated enterocolitis should rather be considered a spectrum covering a wide range of histological patterns, within which variation overlap and/or evolution is possible over time. Again, adequate clinical information is crucial in the context of hypogammaglobulinemia, as individual histological patterns of CVID could be mistaken by the pathologist for IBD, microscopic colitis or GVHD (in the context of an allogeneic transplant).

Other histopathological features in CVID-associated enterocolitis include an increased number of foamy histiocytes, potentially resembling Whipple’s disease (however without periodic acid-Schiff positivity), as well as a paucity of plasma cells [7,12,20,22]. According to Kalha et al., granulomas and giant cells are typically not seen in CVID-associated enterocolitis [7]. However, Daniels et al. identified a granuloma in one of their patients and reported an association between CVID and granulomatous disease (more specifically sarcoidosis) in up to 10% of patients [12]. In this study, a granuloma was seen in one patient’s ileal biopsies, contributing to the CD-like presentation. We also report foveolar gastric metaplasia in the duodenal biopsies of one patient, a histopathological feature that has not yet been reported to be associated with CVID. However, foveolar gastric metaplasia is a nonspecific feature that is often seen in duodenal biopsies of immunocompetent patients in the context of peptic duodenitis, Helicobacter pylori (H. pylori) infection, non-steroidal anti-inflammatory drug (NSAID) intake or (rarely) celiac disease [42,43]. Hence, it is likely that this finding may be attributable to a cause other than CVID.

Plasma cells are abundant in the LP of the normal GI tract, except for the esophagus and stomach, where they are often lacking [20]. In CVID, however, there is often a paucity of plasma cells in the GI tract [7,10,12,20,22,44]. In this study, 5/7 patients had a paucity of plasma cells in at least one sampled part of the bowel. In these cases, only a handful of plasma cells scattered throughout the LP were identified with MUM1 and CD138 IHC. Presence of plasma cells in CVID patients may vary between the duodenum, the small intestine and the colon [20], which was also the case in this study where a paucity of plasma cells was observed in 5/7 patients, though only focally in one part of the gut in 3/5 patients. Biagi et al. and Daniels et al. both suggest that a paucity of plasma cells in GI biopsies is highly suggestive of CVID, but CVID cannot be ruled out in cases with normal plasma cell counts [12,20,38,45,46]. It must be noted that a paucity of plasma cells is not specific to CVID and may also be seen in other PIDs such as X-linked agammaglobulinemia [20,22]. The pathologist should always carefully look for presence of plasma cells (if needed with the help of IHC) in the LP of gastrointestinal biopsies. However, special attention should be paid, since a paucity of plasma cells can be focal, and this feature is easily overlooked. A paucity of plasma cells should always be reported to the clinician, which in turn should rule out immunodeficiency.

In CVID patients, a celiac disease-like enteropathy has been reported to be the most frequent pathologic finding in the small intestine [1,7,12,20,22,26]. However, cases of co-existing CVID and true celiac disease have been reported [12,45,47]. Jørgensen et al. reported that the celiac disease-like enteropathy seen in CVID and true celiac disease are in fact different disease entities, based on gene-expression studies and human leukocyte antigen (HLA) typing [46]. Differentiating these two disease entities is challenging, since serologic tests are unreliable because of the impaired antibody response in these patients [12,46]. Moreover, the HLA profile in CVID patients with a celiac-like disease is inconsistent (although some authors report that HLA typing can be useful to exclude celiac disease in CVID patients) [45], as is the response to a gluten-free diet, further complicating the diagnosis of true celiac disease in these patients [45,46,48]. Although data on the (clinical and histological) response to gluten withdrawal are conflicting, and some authors report that patients with a CVID-associated enteropathy respond to a gluten-free diet in up to 50% of cases [1,49,50], CVID patients generally do not respond to a gluten-free diet [22,46], as was observed in one of our patients, and the only criterion to date to confirm the diagnosis of celiac disease in CVID patients is still the histological response to gluten withdrawal [8,45,48,51]. CVID-associated enteropathy is often characterized by villous blunting and/or an increased number of IELs, as seen in celiac disease. However, in contrast to true celiac disease, there is often a paucity of plasma cells and there is usually no crypt hyperplasia [1,7,10,12,22,46]. The prevalence of villous atrophy and intraepithelial lymphocytosis shows wide variations in the literature, with the prevalence of villous atrophy ranging from 24% to 51% and the prevalence of intraepithelial lymphocytosis ranging from 17% to 76% [8,12,20,52], which is in line with our findings: 4/7 patients had duodenal intraepithelial lymphocytosis, with two of these patients also having villous atrophy. However, both patients also had a paucity of plasma cells, and one of both patients had no obvious crypt hyperplasia, which helped to distinguish from true celiac disease. Nevertheless, we report crypt hyperplasia in one patient with a celiac disease-like pattern, complicating the differential diagnosis between a celiac disease-like CVID-associated enteropathy and true celiac disease. However, this patient had a paucity of plasma cells in the duodenum and neither clinically nor histologically responded to gluten withdrawal, which argues for a celiac-like enteropathy. Both the pathologist and gastroenterologist should be aware of the existence of a celiac disease-like pattern in CVID-associated enteropathy and the possible coexistence of CVID and true celiac disease. To help differentiate between these two entities, the pathologist should pay close attention to the presence/absence of plasma cells and crypt hyperplasia.

CVID is known to predispose to infections [53], and a subset of CVID patients are more at risk for opportunistic infections because of both an impaired humoral (B-cell dependent) immunity and an impaired cell-mediated (T-cell dependent) immunity, with the latter possibly resulting from immunosuppressive therapy, infection or as part of the pathogenesis of CVID [12,26]. Although CMV enterocolitis is reported in CVID patients [12,17], this was not observed in this study. Even though some authors report no increased risk of clinical CMV disease in CVID patients [12,54,55], serology may be falsely negative in the setting of hypogammaglobulinemia, and biopsy samples should be carefully examined to exclude CMV [12,55]. Giardia lamblia is the most common infection and cause of malabsorption in the small intestine [7]. It occurs in 30% to 64% of patients with CVID [7,12,22,26]. Histologic features vary from mild abnormal villous architecture to total villous atrophy, resulting in malabsorption and steatorrhea [1,7,22]. Giardiasis is also known to mimic celiac disease and must always be ruled out in cases clinically suspicious for celiac disease [7]. Another common feature associated with giardiasis is NLH [20]. In this study, one patient was found to have giardiasis. However, this patient had neither obvious villous blunting nor NLH. Although the prevalence of Giardia has decreased over the years because of IVIg therapy [23], these organisms should nonetheless be actively sought for by the pathologist in any duodenal biopsy from a patient with CVID [12,22].

Many CVID patients are reported to have NLH [1,12,20,22,52,56,57]. Sampsa et al. even reported NLH to be the most observed histological feature in the bowel of CVID patients [21]. NLH is often characterized by multiple small nodules in the GI tract ranging from 2 to 10 mm in diameter, mostly distributed in the small intestine [58], and often observed during endoscopy or capsule enteroscopy [59]. LH may also be present microscopically in the absence of gross intestinal nodularity [1]. Histopathology is characterized by the presence of prominent lymphoid aggregates in the LP or submucosa with hyperplastic and (mitotically active) germinal centers, surrounded by well-defined mantle zones [56]. NLH is thought to result from failed B-cell follicle formation [7]. Some authors suggest that it is a compensatory response for the antibody deficiency in CVID [10]. In this study, NLH was seen in 2/7 patients, which is in line with other reports: approximately 20% of patients with CVID are reported to have NLH [58], with prevalences ranging from 8% to 81% [1,7,20,49,55,56]. NLH is not specific to immunodeficient patients and may also be seen in normal individuals, as well as in the context of giardiasis [20]. However, NLH in CVID is reported to be more generalized, with involvement of the proximal small intestine, the terminal ileum and the proximal colon [1,58], which was not observed in this study.

Although there are some reports on the development of lymphoma in patients with NLH, it is not clear whether NLH is a predisposing factor in the development of intestinal lymphoma [1,4,10,12,17,22]. On the other hand, however, CVID is known to predispose to the development of lymphoproliferative disorders and lymphomas, as well as other malignancies, with the latter two being the most common causes of death in patients with CVID [7,8]. CVID patients are 5 times more likely to develop malignancies and 30 times more likely to develop lymphomas [7,8,60]. These include mostly intermediate- to high-grade extranodal non-Hodgkin B-cell lymphomas, with a frequent association with Epstein–Barr virus (EBV) infection [7,8,22]. The latter was also observed in this study, where one patient had eventually developed Burkitt lymphoma. Other malignancies reported in the context of CVID include carcinomas of the breast, uterus, brain and most frequently the gastrointestinal tract [7,8,12]. There is reportedly a 50-fold risk of gastric carcinoma in CVID patients, which can be attributed to the high prevalence of H. pylori gastritis in these patients [1,7,12,60]. Since patients with CVID are at risk of developing malignancies, alarming symptoms including anorexia, excessive weight loss, and lymphadenopathy should always encourage evaluation for malignancy in these patients [7].

This study has some limitations: due to the retrospective nature of this study, not all data were collected at the same time point. Not all patients had complete medical records available since some patients were previously monitored in another hospital. Only biopsy samples available at our institution were reviewed. Moreover, histopathological features can change under therapy, which was not considered in this study. We recognize that our small sample size makes it difficult to draw conclusions and necessitates further research to confirm our findings in larger cohorts.

## 5. Conclusions

In this study, we present seven CVID patients with GI manifestations, highlighting histopathological clues for CVID in intestinal biopsies and emphasizing diagnostic pitfalls for the pathologist and gastroenterologist. GI manifestations are a major cause of morbidity in CVID and can be the initial presentation [7,18,22]. Hence, immunodeficiencies should be considered in patients with chronic GI problems [1], and immunosuppressive medications should be administered carefully [8,12,26,39,40]. Care must be taken when a diagnosis of IBD or celiac disease is considered in the context of CVID, since the clinical presentation and histology often mimic other GI conditions, such as IBD, microscopic colitis, celiac disease and/or GVHD [1,7,8,12,19,20,21,22,23,24,25,26], hence the importance of providing adequate clinical information with mention of known or suspected CVID. Some histological features are atypical of the aforementioned established entities and may indicate CVID, as may overlapping and/or changing histological patterns and/or a paucity of plasma cells in part of the gut [7,10,12,20,22], the latter of which is often only focal and easily overlooked when not carefully searched for. However, additional research is needed to confirm these findings in larger cohorts. Last but not least, GI infections should always be ruled out in patients with CVID [12].

Diagnosis of CVID remains challenging and is often delayed, potentially resulting in lethal complications. Awareness of the heterogenous clinical presentation and histopathological indicators of CVID may lead to improved diagnosis and earlier treatment, thereby avoiding these potentially life-threatening complications.

## Figures and Tables

**Table 1:** Percentage of patients showing histological abnormalities.

Histopathological Findings	Duodenum	Ileum	Colon
Normal	5/7 (71%)	3/7 (43%)	5/7 (71%)
Villous atrophy	2/7 (29%)	0/7 (0%)	-
Duodenal crypt hyperplasia	1/7 (14%)	-	-
Increased IELs (any degree)	4/7 (57%)	6/7 (86%)	3/7 (43%)
*Severely increased IELs*	*2/4 (50%)*	*1/6 (17%) (=lymphocytic sprue-like)*	*2/3 (67%) (=lymphocytic colitis-like)*
*Villous atrophy*	*2/4 (50%) (=celiac disease-like)*	*0/6 (0%)*	*-*
*Crypt hyperplasia*	*1/4 (50%) (=celiac disease-like)*	*-*	*-*
Low number of PCs	4/7 (57%)	2/7 (29%)	3/7 (43%)
Crypt distortion	-	1/7 (14%)	1/7 (14%)
Active inflammation	0/7 (0%)	3/7 (43%)	6/7 (86%)
*Crypt distortion*	*-*	*1/3 (33%) (=IBD-like)*	*1/6 (17%) (=IBD-like)*
Granulomas	0/7 (0%)	1/7 (14%) *(=IBD-like)*	0/7 (0%)
Lymphoid hyperplasia	0/7 (0%)	3/7 (43%)	0/7 (0%)
Increased apoptosis	0/7 (0%)	0/7 (0%)	2/7 (29%) (=GVHD-like)
Thickened subepithelial collagen layer	0/7 (0%)	0/7 (0%)	2/7 (29%) (=collagenous colitis-like)
Foveolar gastric metaplasia	1/7 (14%)	-	-
Pyloric gland metaplasia in the ileum/colon	-	2/7 (29%) (=IBD-like)	0/7 (0%)
Giardiasis	1/7 (14%)	1/7 (14%)	-

IELs = intraepithelial lymphocytes; PCs = plasma cells; IBD = inflammatory bowel disease; GVHD = graft-versus-host disease.

## Data Availability

The original contributions presented in this study are included in the article. Further inquiries can be directed to the corresponding author.

## Abbreviations

The following abbreviations are used in this manuscript:

MDPI	Multidisciplinary Digital Publishing Institute
DOAJ	directory of open access journals
TLA	three letter acronym
LD	linear dichroism
CVID	common variable immunodeficiency disorder
IBD	inflammatory bowel disease
GVHD	graft-versus-host disease
PID	primary immunodeficiency
Ig	immunoglobulin
SD	standard deviation
GI	gastrointestinal
CD	Crohn’s disease
UC	ulcerative colitis
IVIg	intravenous immunoglobulin
SCIg	subcutaneous immunoglobulin
CID	combined immunodeficiency disorder
IQR	interquartile range
LH	lymphoid hyperplasia
IELs	intraepithelial lymphocytes
HE	hematoxylin and eosin
IHC	immunohistochemistry
LP	lamina propria
MUM1	multiple myeloma oncogene-1
CMV	cytomegalovirus
NLH	nodular lymphoid hyperplasia
IBS	irritable bowel syndrome
AIHA	autoimmune hemolytic anemia
ITP	idiopathic thrombocytopenic purpura
EDS	Ehlers–Danlos syndrome
*H. Pylori*	*Helicobacter pylori*
EBV	Epstein–Barr virus
n°	number
Pat.	patient
y	years
CI	clinical information
M	male
F	female
PCs	plasma cells
MP	metaplasia
N/A	not available

## Appendix A

**Table A1:** Clinical characteristics and bowel histopathology of 7 CVID patients, listed in chronological order.

Pat. n°	Age (y)	Sex	Age at Diagnosis (y)	GI Presentation	Other Clinical Manifestations	Biopsy n°	Histopathology				Endoscopy Findings	CI
							Duodenum	Ileum	Colon	Pattern(s)		
1	63	M	53	CD, blood in stool, chronic watery diarrhea, abdominal discomfort, celiac disease-like presentation	Respiratory infections, Burkitt lymphoma	1	Normal	Normal	-	Normal	N/A	(0/2)
						2	-	Active inflammation, granuloma, few PCs	Active inflammation, few PCs	IBD-like	Ileum: NLH; Colon: normal	0/1
						3	-	Normal	IELs ++, increased apoptosis, few PCs	Lymphocytic colitis-like, GVHD-like	Ileum: NLH; Colon: normal	0/1
						4	Normal	LH	Active inflammation, IELs ++, thickened collagen layer ++	Collagenous colitis-like	Duodenum and colon: normal; Ileum: NLH	0/2
						5	IELs +	-	-	No specific pattern	Duodenum: nodular aspect	1/1
						6	IELs ++, villous atrophy, crypt hyperplasia, few PCs	IELs +	Normal	Celiac disease-like	Duodenum: atrophic aspect; Ileum and colon: normal	2/2
						7	IELs ++, villous atrophy, few PCs	-	-	Celiac disease-like	Duodenum: normal	1/2
2	49	M	44	Diarrhea, abdominal discomfort	Respiratory infections, lymphadenopathy	1	Normal	Normal	Normal	Normal	Duodenum, ileum and colon: normal	1/2
						2	Foveolar gastric MP, few PCs	IELs +	Active inflammation	Acute colitis-like	Duodenum, ileum and colon: normal	0/2
3	56	F	47	Constipation, obstruction, abdominal discomfort, lactose intolerance, IBS, diarrhea, blood in stool	EDS, respiratory infections	1	-	IELs +	Normal	No specific pattern	Ileum: normal; Colon: minor ulcera	0/1
						2	-	Active inflammation, pyloric gland MP	Normal	IBD-like	Ileum and colon: minor ulcera	1/1
						3	-	Normal	Active inflammation	Acute colitis-like	Ileum and colon: erythema, minor ulcera	1/1
						4	-	IELs+	-	No specific pattern	Ileum: normal	0/1
						5	Normal	-	Normal	Normal	Duodenum: normal; Colon: minor ulcera	0/2
4	43	F	29	Diarrhea, abdominal discomfort	Respiratory infections, mastitis	1	IELs +, giardiasis	IELs +, giardiasis	IELs ++, few PCs	Lymphocytic colitis-like	Duodenum and colon: normal; Ileum: NLH	1/2
5	37	F	28	Diarrhea, malabsorption	Respiratory infections, skin infections, AIHA, ITP	1	Villous atrophy, IELs ++, few PCs	IELs ++, few PCs	Active inflammation, few PCs	Celiac disease-like, lymphocytic sprue-like, acute colitis-like	Duodenum and colon: normal; Ileum: atrophic aspect	0/2
6	20	M	11	CD, blood in stool, diarrhea, abdominal discomfort, obstruction	Respiratory infections	1	Normal	Active inflammation, crypt distortion	Normal	IBD-like	Duodenum: normal; Ileum: erythema, fibrin plaques; Colon: minor ulcera	(0/1)
						2	-	LH	Active inflammation, crypt distortion	IBD-like	Ileum and colon: normal	0/1
						3	-	Active inflammation, crypt distortion, pyloric gland MP	-	IBD-like	N/A	0/1
						4	Normal	Active inflammation, pyloric gland MP	Active inflammation	IBD-like	Duodenum: normal; Ileum and colon: erythema, ulcera	0/1
						5	-	Active inflammation, crypt distortion	Active inflammation	IBD-like	Ileum: ulcera; Colon: normal	1/1
						6	-	Crypt distortion	Active inflammation, crypt distortion	IBD-like	Ileum and colon: normal	0/1
7	53	F	46	Diarrhea, abdominal discomfort, lactose intolerance, NLH	Spondylo-arthropathy, chondrosarcoma, respiratory infections	1	Normal	IELs +, LH	-	No specific pattern	N/A	(0/1)
						2	Normal	-	-	Normal	N/A	(0/1)
						3	-	IELs +, LH	Normal	No specific pattern	N/A	(0/1)
						4	IELs +	-	IELs +, increased apoptosis, active inflammation	GVHD-like	Duodenum, ileum and colon: normal	(0/1)
						5	-	-	Thickened collagen layer ++	Collagenous colitis-like	Colon: erythema	(0/1)
						6	Few PCs	IELs +, LH	IELs +	No specific pattern	Duodenum and colon: normal; Ileum: NLH	1/2
						7	Normal	-	-	Normal	Duodenum: normal	0/1
						8	-	IELs +	IELs +, active inflammation	Acute colitis-like	Ileum: normal; Colon: edema	1/1
						9	Few PCs	-	-	No specific pattern	Duodenum: normal	1/1

Pat. = patient; n° = number; y = years; GI = gastrointestinal; CI = clinical information, shown as the ratio of pathology requests mentioning known/suspected CVID (results in parentheses mean this information was not on file in medical records and there was no diagnosis of CVID at that time); M = male; F = female; CD = Crohn’s disease-like presentation; NLH = nodular lymphoid hyperplasia upon endoscopy; IELs = intraepithelial lymphocytes, graded + (mild to moderate increase, >5 IELs/100 surface epithelial cells) or ++ (severe increase, >20 IELs/100 surface epithelial cells); a thickened collagen layer was graded + (≥3 µm) or ++ (>10 µm); LH = lymphoid hyperplasia; PCs = plasma cells; MP = metaplasia; IBS = irritable bowel syndrome-like presentation; EDS = Ehlers–Danlos syndrome; AIHA = autoimmune hemolytic anemia; ITP = idiopathic thrombocytopenic purpura; N/A = no data available.
